# Sex-sensitive and insensitive endocrine responses and infradian changes while trekking in the Himalayas

**DOI:** 10.1007/s00424-026-03166-z

**Published:** 2026-04-08

**Authors:** Danilo Bondi, Sofia Bonan, Simone Cantarelli, Francesca Ietta, Ilenia Casini, Alberto Taverna, Riccardo Rua, Maria Teresa Guagnano, Arcangelo Barbonetti, Suwas Bhandari, Ashish Shrestha, Anna Maria Aloisi, Vittore Verratti

**Affiliations:** 1 Department of Neurosciences, Imaging and Clinical Sciences, University “G. d’Annunzi” Chieti - Pescara, Via dei Vestini, Chieti, 31 - 66100 Italy; 2 Department of Medicine and Ageing Sciences, University “G. d’Annunzio” Chieti - Pescara, Chieti, Italy; 3 Department of Medicine, Surgery and Neuroscience, University of Siena, Siena, Italy; 4 Medical specialty school of Anaesthesia, resuscitation, intensive care and pain management, University of Trento, Trento, Italy; 5 Medical specialty school of Anaesthesia, resuscitation, intensive care and pain management, University of Torino, Torino, Italy; 6 Department of Life, Health and Environmental Sciences, University of L’Aquila, L’Aquila, Italy; 7 School of International Studies, Wenzhou Medical University, Wenzhou, China; 8 Department of Pulmonary and Critical care, Sun Yat-Sen University, Guangzhou, China; 9 Department of Science, University “G. d’Annunzio” Chieti - Pescara, Chieti, Italy; 10 Italian Society of Mountain Medicine, Padova, Italy; 11 Center for Disability, Rehabilitation, and Sports Medicine (CARES), University “G. D’Annunzio” Chieti - Pescara, Chieti, Italy

**Keywords:** Menstrual cycle length, Stress hormones, Hypothalamic-pituitary-gonadal axis, Hypobaric hypoxia, High-altitude

## Abstract

Various studies have explored the effects of altitude hypoxia on hormonal changes. However, most research has been conducted on small samples and focused solely on male subjects. This project aimed to examine the effects of high-altitude trekking on the hypothalamic-pituitary-gonadal axis and the hormonal stress response, with particular attention to potential gender differences in hormonal fluctuations. A Himalayan expedition was conducted with 21 volunteers (12 men, 9 women), aged 43 ± 15 years, with a BMI of 24.2 ± 3.70 kg/m². Participants took a 250 mg pill of acetazolamide once daily. Blood samples were collected before, during, and after the ascent to a Pyramid Laboratory - Observatory (altitude approximately 5,000 m above sea level). Five females were premenopausal, all in the follicular phase of their menstrual cycle. A longer cycle duration was observed in all of them in response to the high-altitude expedition. Gonadotropin levels remained unchanged in both sexes. Differential trends according to sex emerged for: 17β-estradiol, as females showed a decrease from low to high altitude, while males’ reductions were delayed; prolactin, whose decline at high altitude was more pronounced in females; progesterone, as males exhibited higher values at high altitude than at low altitude, whereas the overall trend was flat in females. The dysregulation of the endocrine system at high altitude is subclinical and reversible in both sexes, at least up to 5,000 m a.s.l. The significant prolongation of the menstrual cycle in response to hypoxia warrants further detailed analyses.

## Highlights

• altitude hypoxia leads to longer menstrual cycles in humans.

• rather than cortisol, prolactin is a highly responsive hormone in both sexes to the combined stressors of trekking and altitude hypoxia.

• prolactin, progesterone ed estradiol exhibited a sex-sensitive response.

## Introduction

The collective evidence suggests that hypoxia should not be regarded merely as an environmental stimulus, but rather as a determinant capable of hierarchically reorganising physiological control systems. From this perspective, high-altitude exposure may be viewed as a model of integrative plasticity, in which the interplay among environmental, neuroendocrine, and metabolic factors reveals the intrinsically dynamic nature of bodily systems. Conditions such as chronic obstructive pulmonary disease, obstructive sleep apnoea, diabetes mellitus, severe anaemia, and chronic respiratory failure share a common hypoxic substrate in which endocrine adaptation gradually loses its reversibility. Within this context, the physiological experience of high altitude provides a unique natural paradigm for understanding how systemic hypoxia reshapes the priorities of human endocrine regulation, bridging the gap between environmental physiology and clinical pathophysiology. Various studies have been conducted in the last decades to investigate the effects of altitude hypoxia on hormonal changes. However, most studies have been conducted on small samples, limited to male subjects, and have been conducted primarily at altitudes not exceeding 5000 m, whether real or simulated [1–6].

In a recent study carried out in Mount Himlung Himal, Nepal, a 6-point analysis was carried out across baseline (550 m), altitude base camps (4844 m and 6022 m) and peak altitude of expedition (7050) m [7]; results showed that hypothalamic–pituitary-adrenal (HPA) and thyroidal (HPT) axes, as well as prolactin secretion, resulted to be activated by hypoxia, while in male hypothalamic–pituitary-gonadal (HPG) axis resulted inhibited. Variation of testosterone was SaO_2_-dependent, and variation of prolactin was sex -dependent; variation of cortisol, prolactin, Follicle Stimulating Hormone (FSH) and Luteinizing Hormone (LH) were not SaO_2_ or PaO_2_ dependent; acclimatisation at 4844 m led to normalisation of adrenal and gonadal but not of thyroid axes.

High altitude trek can affect the hormonal axes by suppressing the HPG axis at the gonadal level [2]. The inhibitory hypoxia-induced effects on HPG were ascribed to gonadal impairment; however, it was reported that a significant altitude-induced decline in Gonadotropin-Releasing Hormone (GnRH) and a consequent decrement in FSH and LH were not accompanied by changes in testosterone secretion, which remained relatively stable until very high altitudes [8]. A depression of the gonadal axis was also reported from a Himalayan expedition to Kanchenjunga, where lower total testosterone and 17-β-estradiol levels were observed after the expedition, which reversed during the follow-up period.

Most studies have focused on males due to the cycle-related fluctuations in female gonadotrophins [9]. However, the study of female subjects is critical because of the cardiovascular and respiratory sex differences found in hypoxia, responses shown to be sensitive to sex hormone fluctuations [5]. These differences may lead to differential symptom occurrence, as a meta-analysis reported a higher prevalence of acute mountain sickness (AMS) in females than in males [10], despite evidence that females are generally less susceptible to hypoxia-associated cellular and tissue damage [11].

In another study report from Mount Himlung Himal, researchers studied the short-term hormonal fluctuation in females by sampling blood every 10–20 min for a total interval of 6 h at low altitude, 4,800 m and peak altitude of 6,050 m, reporting that pulse decrement in response to high altitude was not permanent, and that the increase in hormone concentrations found with altitude, can be attributed to a marked rise in mass per pulse, since pulse frequency decreased; in particular, pulse frequencies of both prolactin and cortisol were reduced from baseline to 4,800 m, but then increased with acclimatization from 4,800 m to 6,050 m [9]. The altered timing and magnitude of adenohypophyseal release are unlikely to depend on modifications in hypothalamic drive, since hypothalamic responsiveness to hypoxia does not appear to be significantly affected [12]. The marginal involvement of the hypothalamic level actually offers greater consistency in the study, given that measuring hypothalamic factor release is very difficult, whereas measuring pituitary and gonadal hormones of the HPG axis is much more reliable.

This project aimed to investigate the effects of high-altitude expedition on the HPG axis and hormonal stress response, with particular attention to potential gender differences in hormonal fluctuations. The study was designed to evaluate fluctuations in female hormones without altering the duration or timing of the ovarian cycle through drug treatments.

## Methods

### Design of the study

**Fig. 1:**
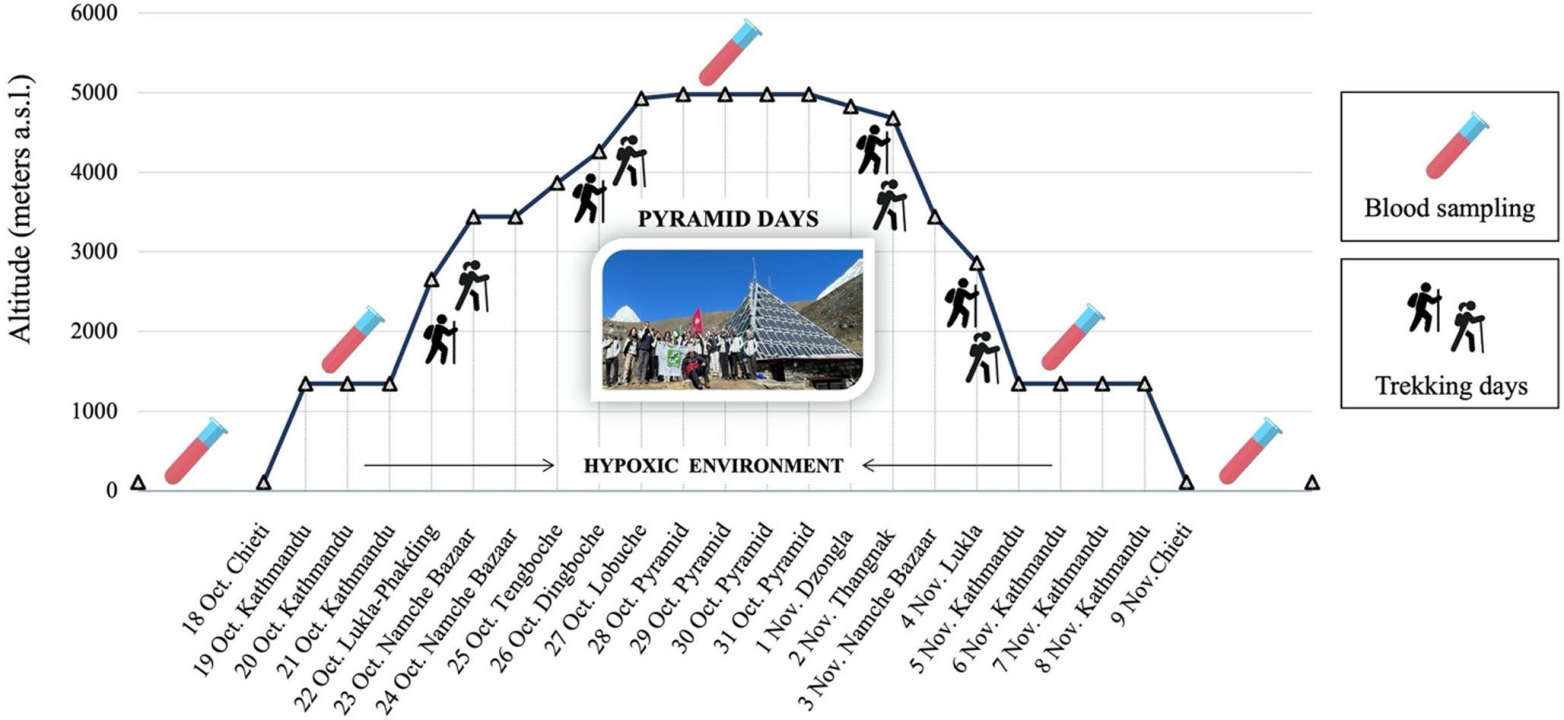
Study design and altimetric plan of the “Lobuche Peak-Pyramid Exploration & Physiology” Himalayan expedition

Both male and female participants took part in the international expedition “Lobuche Peak - Pyramid Exploration & Physiology,” conducted in the Sagaramāthā (Mount Everest) National Park, Nepal, from October 19 to November 9, 2022. Inclusion criteria were: age > 18 years and signing informed consent; exclusion criteria were: diagnosis of cardiovascular disease; diagnosis of chronic obstructive pulmonary disease; diagnosis of psychiatric disease; diagnosis of neurological diseases; diagnosis of respiratory failure; uncontrolled hypertension. All procedures performed in studies involving human participants adhered to the ethical standards of the institutional and/or national research committee and to the 1964 Declaration of Helsinki and its later amendments, or to comparable ethical standards. This work represented an ancillary part of a study approved by the local Institutional Review Committee. Once included in the study, all participants signed the informed consent and agreed to the trekking and blood collecting program, as reported in detail in Fig. 1:

- Chieti 1: baseline level, at Chieti University and “San Salvatore” Hospital in L’Aquila, Italy, at altitudes of 50–700 m a.s.l.
- Kat 1: Upon arrival, 1–2 days before the start of trekking. Omkaar Polyclinic, which is located in Kathmandu, Nepal, at an approximate altitude of 1,400 m (LA, low altitude).
- Pyr-International Pyramid Laboratory in the Khumbu Valley, the peak altitude reached during the expedition, at approximately 5,000 m; it was reached after 6 days of trekking (HA, high altitude); the descent from Pyramid started following a five-day sojourn and was completed within a span of four days.
- Kat 2: at the end of trekking.
- Chieti 2: follow-up, once returned to Italy.

Meal and sleep times remained similar during the stay in the Pyramid compared to those during the ascent and descent phases. The expedition involved a day of sampling, conducted two to three days after arrival at each research site to avoid the acute effects of ascent and descent. Young females were asked to report their cycle day. Sampling occurred two to three days after the arrival at each research site.

### Participants

As reported in Table 1, the group of trekkers consisted of 21 Italian travellers of lowlander origin who lived at low altitude, 12 of whom were male and 9 of whom were female, with an average age of 43 ± 15 years, and an average BMI of 24.2 ± 3.7 kg/m² (see Table 1). Only one male participant reported an endocrine disease, i.e., autoimmune thyroiditis. Of the nine females involved, three were post-menopausal, and one was previously subjected to a hysterectomy with bilateral oophorectomy. All of those had their last menstruation at the start or in the first phase of the expedition and were therefore in the follicular phase of the initial ovarian cycle during their stay at the peak altitude. The other five younger females had cycle lengths ranging from 28 to 40 days; none were professional athletes.

**Table 1:** Characteristics of the study group. Data are expressed as mean ± SD. BMI: body mass index

	*N*.	Age (years)	BMI (kg/m^2^)	Note
Males	12	46.92 ± 14.84	25.72 ± 2.51	1 autoimmune thyroiditis
Pre-menopausal females	5	27.80 ± 2.28	20.78 ± 2.19	/
Non-fertile females	4	59.25 ± 7.18	26.05 ± 3.94	1 past hysterectomy

As planned, all trekking phases were respected, and all subjects concluded the trekking experience in good health. To facilitate acclimatisation, all participants took 250 mg of acetazolamide once daily during the ascent.

### Procedures

All the tests were conducted in the morning. Blood was collected in tubes with or without an anticoagulant, depending on the hormone assay. Blood was collected via antecubital vein sampling and subsequently centrifuged to separate the liquid fraction, which was then transferred to test tubes, stored and transported for subsequent analysis. The samples were stored with multiple levels of insulation to protect it from potential external contamination. The cold chain was managed with attention to details; in particular, the samples were maintained at the appropriate temperature by the use of refrigerators at each station in the chain and refrigerated boxes during transport to each station. In consideration of the context of the field study, it was not feasible to maintain an exact temperature throughout the chain. However, through the implementation of thermometer monitoring, the temperature was recorded as approximately − 5 °C. The following hormones were considered: FSH, LH, Testosterone, Estradiol, Progesterone, Prolactin, and Cortisol. Hormonal concentrations were quantified through an automatic immunoassay analyzer (Tosoh Bioscience Srl, Italy), with the following kits of ST AIA-PACK series: PRL (for Prolactin), TESTOSTERON (for Testosterone), CORT (for Cortisol), IE2 (for 17β-estradiol), FSH (for Follicle Stimulating Hormone), LH II (Luteinizing Hormone), and PROG (for Progesterone).

### Statistical analyses

The normality of the residuals was assessed using the Kolmogorov–Smirnov test and observing Q–Q plots. In light of the findings, in instances where the normality of the residuals or the visual assessment of the conditional residuals line failed to ensure adherence to the assumptions, the elimination of one or more subjects was subjected to independent evaluation for each analytical procedure. This removal was based on values that were physiologically outside the baseline ranges or on the presence of residuals that were very distant from the mean. Subsequent to each removal, a re-examination of the assumptions was conducted. Then, repeated-measures analyses were then conducted using a series of mixed linear models fitted by restricted maximum likelihood (REML), with time and sex as fixed factors and individuals as the random factor. Following Mauchly’s test for sphericity, Greenhouse–Geisser’s correction was applied if necessary. To calculate degrees of freedom, Satterthwaite’s method was used. Post hoc analyses were performed with multiple comparisons adjusted using Tukey’s method. All studies and graphical representations were performed using Prism Version 10 (GraphPad Software, USA).

## Results

**Fig. 2:**
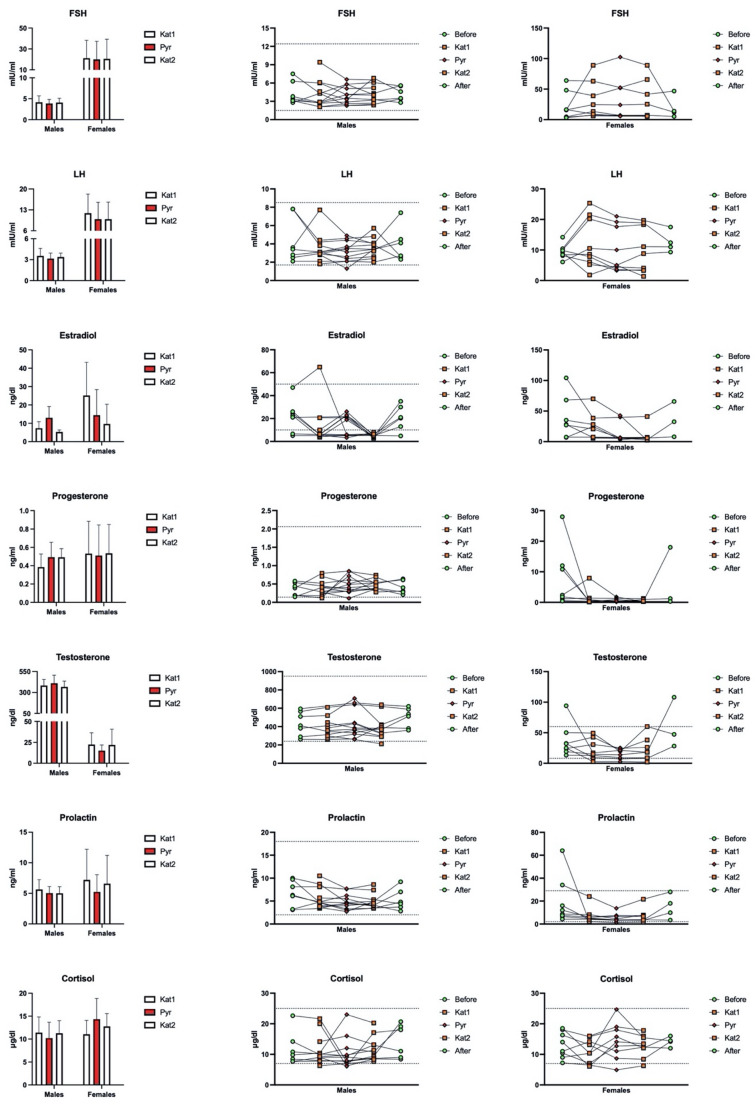
Summary values and individual changes of Follicle Stimulating Hormone (FSH), Luteinizing Hormone (LH), 17β-estradiol, progesterone, testosterone, prolactin, and cortisol across study times

All statistical comparisons are reported in Table 2, and the graphs with summary and individual values are shown in Fig. 2. Table 3 presents summary values for males and females, with the latter further grouped into pre-menopausal and non-fertile stages of life; due to the small sample sizes of the subgroups, the pre-menopausal vs. non-fertile data are reported only as descriptive results, with no additional between-groups comparisons. Analyses show:

- *FSH* - no statistical evidence was found for the altitude travel in the release of FSH nor by interaction of altitude and sex; values resulted in being significantly higher in females than males; in particular, females in non-fertile status clearly show the most significant values; a reduction seemed to occur in pre-menopausal females as a cumulative result of the high-altitude trekking (Table 3);
- *LH* - results mirror those of FSH, with no significant effect of sex, altitude or sex × altitude, greater values in females than males (particularly high in non-fertile age), and a possible reduction in pre-menopausal females as a cumulative result of the high-altitude trekking (Table 3);
- *17β-estradiol* - values were significantly influenced by altitude and sex (Table 2), indeed as shown in Fig. 2 the lowest values were reached at the end of the expedition; this effect was sex-dependent, as females (both pre-menopausal and non-fertile, Table 3) exhibited a decrease from low to high altitude, while males’ reduction were delayed to the end of expedition.
- *Testosterone* - values did not significantly change across study phases; the trend was only slightly and not significantly different by sex; values were relatively less heterogeneous among males than females (Fig. 2); males exhibited massively higher values than females, and the lowest values were observed in non-fertile females (Table 3).
- *Prolactin* - values resulted in differentially affected by altitude in the two sexes; indeed, the overall decline at high altitude was more pronounced in females with respect to males; moreover, a partial recovery was observed only in females (and particularly in pre-menopausal females, Table 3) upon return to low altitude.
- *Progesterone* - levels revealed a significant interaction Sex x Altitude (Table 2), as males exhibited greater values at high altitude than low altitude, whereas the average trend was almost flat in females; pre-menopausal females seemed to show a reduction in response to high-altitude trek (Table 3), while non-fertile females did not.
- *Cortisol*: the changes were largely heterogeneous between individuals; as shown in Fig. 2, values remained in acceptable ranges across study times; pre-menopausal females seem to exhibit slightly higher values than the other groups (Table 3).

**Table 2:** Statistical results from mixed linear models fitted by restricted maximum likelihood, with time and sex as fixed factors and individuals as the random factor. Comparisosn by sex refers to females vs. males; comparisons by altitude refer to before vs. Kathmandu 1 vs. Pyramid vs. Kathmandu 2 vs. after. The female group encompasses both pre-menopausal and non-fertile women; for further details concerning the two groups separately, please refer to Table 3

	Sex		Altitude		Sex × altitude	
Hormone	*p* value	F (DFn, DFd)	*p* value	F (DFn, DFd)	*p* value	F (DFn, DFd)
FSH	0.017	F(1,19) = 6.874	0.687	F(4,55) = 0.5674	0.703	F(4,55) = 0.545
LH	0.002	F(1,19) = 12.77	0.505	F(4,58) = 0.841	0.314	F(4,58) = 1.216
Progesterone	0.030	F(1,19) = 5.531	0.177	F(4,53) = 1.644	0.013	F(4,53) = 3.504
17β-estradiol	0.074	F(1,18) = 3.587	< 0.001	F(4,52) = 7.957	0.043	F(4,52) = 2.662
Testosterone	< 0.001	F(1,19) = 78.54	0.538	F(4,47) = 0.733	0.166	F(4,47) = 1.768
Prolactin	0.100	F(1,19) = 2.984	< 0.001	F(4,57) = 9.560	0.003	F(4,57) = 4.459
Cortisol	0.394	F(1,19) = 0.760	0.406	F(4,58) = 1.017	0.322	F(4,58) = 1.198

*FSH* follicle-stimulating hormone, *LH* luteinizing hormon, *DF* degrees of freedom

**Table 3:** Summary values of hormones at three study times in the three different groups (males, pre-menopausal females and non-fertile females)

	Males (*n* = 12)			Pre-menopausal females (*n* = 5)			Non-fertile females (*n* = 4)		
Hormone	Kat1	Pyr	Kat2	Kat1	Pyr	Kat2	Kat1	Pyr	Kat2
FSH (mlU/ml)	2.90 (2.55)	3.50 (1.85)	4.00 (1.80)	7.40 (1.90)	6.70 (1.00)	6.60 (1.30)	51.50 (34.18)	52.10 (20.20)	53.60 (34.10)
LH (mlU/ml)	3.10 (1.15)	3.10 (1.80)	3.40 (0.80)	6.12 (2.60)	4.40 (1.40)	3.50 (0.80)	20.85 (5.15)	18.40 (5.20)	18.60 (3.18)
Progesterone (ng/ml)	0.31 (0.22)	0.49 (0.36)	0.51 (0.17)	0.68 (1.03)	0.47 (0.91)	0.40 (0.14)	0.33 (0.19)	0.24 (0.27)	0.33 (0.27)
17β-estradiol (ng/dl)	6.10 (2.85)	6.02 (16.10)	5.40 (1.85)	20.70 (31.80)	6.20 (33.90)	6.10 (1.40)	24.90 (10.30)	5.40 (1.80)	6.10 (2.00)
Testosterone (ng/dl)	358.00 (121.60)	370.11 (110.56)	368.00 (91.00)	42.68 (18.64)	22.00 (5.50)	38.00 (33.90)	11.12 (6.38)	8.17 (6.07)	8.58 (4.58)
Prolactin (ng/ml)	4.80 (3.05)	4.60 (1.65)	4.50 (1.05)	6.10 (0.92)	4.50 (2.90)	6.70 (3.30)	4.15 (5.73)	3.35 (3.33)	3.55 (5.20)
Cortisol (µg/dl)	9.28 (3.82)	7.91 (3.67)	9.39 (3.67)	13.12 (8.71)	14.10 (5.29)	13.47 (2.99)	10.39 (1.96)	13.41 (8.50)	12.21 (2.54)

*FSH* follicle-stimulating hormone, *LH* luteinizing hormone, *Kat1* Kathmandu before high-altitude expedition, *Pyr* Pyramid, at high-altitude, *Kat2* Kathmandu, after high-altitude expedition; data are expressed as Median (IQR)

**Fig. 3:**
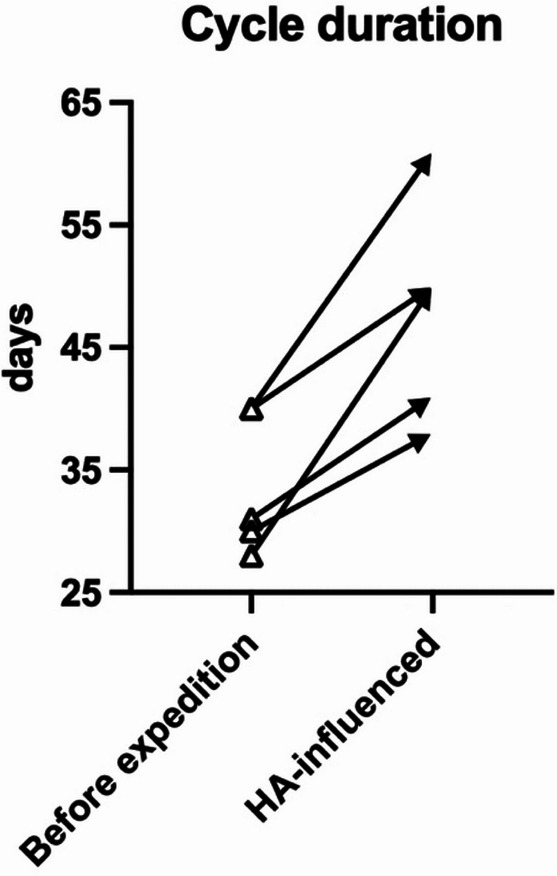
Individual differences in menstrual cycle duration of the five pre-menopausal females before and after the high altitude (HA) expedition

Exposure to high altitude was linked to a mean 14-day extension of the menstrual cycle (*p* = 0.009, t_4_ = 4.72, Hedges’ g = 1.38), as shown in Fig. 3. It should be noted that two females had baseline durations of 40 days, which exceed the normal range.

**Fig. 4:**
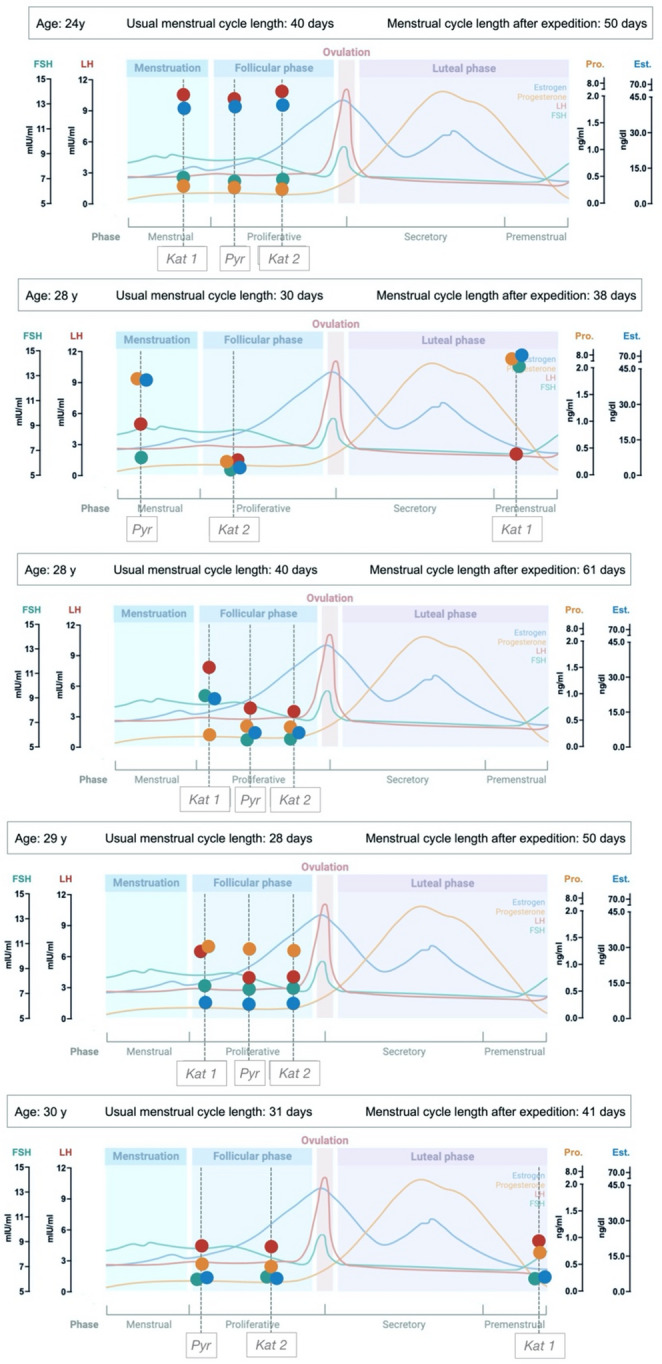
Hormonal values overimposed on a theoretical hormonal fluctuation model during a menstrual cycle; Kat1: Kathmandu before high-altitude expedition; Pyr: Pyramid, at high-altitude; Kat2: Kathmandu, after high-altitude expedition. The background image of the ovarian menstrual cycle was obtained from BioRender. Each measurement for each female is marked with a coloured dot; the four colours identify the hormones estradiol, progesterone, FSH, and LH and correspond to the legend and the titles of the lateral scales

As shown in Fig. 4, all five premenopausal females were in the preovulatory phase during the high-altitude sampling. Hormonal trajectories deviated from theoretical patterns, indicating a slowing of preovulatory dynamics in response to hypoxic exposure.

## Discussion

The primary finding of this study is the observation of hormonal changes in males and females exposed to natural hypoxia during a mountain expedition. Blood samples from premenopausal and non-fertile women, as well as men, were collected to assess sex and stress hormones, demonstrating that altitude/hypoxia can differently influence the hypothalamic-pituitary axis and peripheral hormone secretion.

The literature reports that altitude-induced suppression of the gonadal axis also occurs in females, as the female axis is highly sensitive to stressors and generally responds with dysregulated hypothalamic–pituitary pulsatility. To the best of our knowledge, this is the first study to show that altitude hypoxia leads to longer menstrual cycles in humans, along with irregular changes in hormonal dynamics.

Previous studies reported that exposure to high-altitude affects the corpus luteum’s development and function in female sheep [13] and induces a prolonged cycle in female mice [14]. We hypothesise that putative alterations in pulsatility slow down the infradian rhythms of the female hormonal cycle. This increase in cycle duration may be an acute effect, since neither menstrual cycle length nor regularity was reported to be different in high-altitude natives compared to low-living females [15]. The inconsistency of the follicular phase’s length is what determines the varied duration of the ovarian cycle, while the luteal phase is less variable [16]. Therefore, the fact that all premenopausal females were exposed to altitude hypoxia during the preovulatory phase may have facilitated rhythm relaxation, thereby delaying the LH surge. It should be highlighted than two females had long menstrual cycle length before expedition (i.e., 40 days); those females did not report any factor known to affect cycle length [17]: they did not reported strenuous exercise habits, alcoholic beverages’ or other toxicants’ addiction; their BMI was at the lower threshold for normal weight (18.07 and 18.78 kg/m^2^). In any case, the marked prolongation of the menstrual cycle and the slowing of pre-ovulatory hormonal dynamics suggest an adaptive delay of follicular maturation and ovulation, which could serve as an energy-sparing mechanism in hypoxic environments. These alterations resemble the reversible reproductive suppression described in other stress-related or energy-deficient states.

The impact of high-altitude hypoxia on the hormonal axis does not follow the standard rule of systemic stress response. Rather than the cortisol over-response of the general stress response, the cortisol axis here demonstrated an inter-individual heterogeneous response with a mean flat pattern; by coupling these results with other references [2, 7, 18, 19], we can depict a possible suppression or flat response of the HPA axis at moderate-to-high altitudes, followed by over-activation at altitudes > 5,000 m. Prolactin instead is more responsive to altitude hypoxia: here we observed a decrease at high altitude, as well as in a previous studies on Italian trekkers during a Himalayan expedition two but results are heterogeneous across studies [7, 18, 19]; we interpret prolactin as a highly responsive hormone in both sexes to the combined stressors of physical exercise and hypoxia, that can be reduced until a cumulative stress threshold is achieved. In addition, the potential role of dopamine is noteworthy, as it is a primary inhibitor of prolactin secretion from the pituitary. Contrasting results exist regarding the effects of hypoxia on the dopamine system: e.g., it has been reported that chronic hypoxia reduces the plasma levels of dopamine while increasing the levels of the precursor DOPA [20]; however, the plasma levels of dopamine are greater at high altitude than at low altitude sampling in high-altitude natives [21]. Dopamine inhibiting pituitary prolactin secretion is produced by hypothalamic tuberoinfundibular neurons and moves into the portal system; the dopamine involved in the reward system travels along the mesolimbic pathway; in either case, plasma dopamine would have been an unreliable proxy for activity in the hypothalamic-pituitary portal system or the mesolimbic reward circuit. While only indirectly interpreted via changes in prolactin levels, here we can speculate that the hypoxic stress was not sufficient to disrupt the function of dopamine-releasing neurons, which are typically vulnerable to hypoxia [22]. In contrast, the over-production of all catecholamines, rather than the possible rise of dopamine levels in the reward system, exerted the likely inhibitory over-effect of dopamine on prolactin release through the hypothalamic-pituitary portal system.

Here, prolactin demonstrated a sex-specific response, with fluctuations more pronounced in females than in males. Progesterone also demonstrated a sex-specific reaction, with higher values in males at high altitude but not in females. A previous study at high altitude in premenopausal females reported that progesterone levels remained similar to those observed in the same menstrual phase at sea level before the expedition, with a further apparent decrease after returning to sea level, when all females were in the luteal phase of the cycle. It is worth noting that progesterone is a respiratory stimulant that reduces the activation threshold of the bulbar respiratory centre, thereby increasing its excitability [23]. Indeed, females exhibit a lower hypoxic ventilatory response during the follicular phase, when progesterone levels are lower. The fact that premenopausal females were predominantly in the follicular phase at high altitude may, in part, explain the flat response in females.

Estradiol also exhibited a sex-sensitive response to hypoxia, with the highest values in males but not in females at peak altitude. As far as males are concerned, the hypothesis of an overall male gonadal disruption due to hypoxia [24] may not be the main one in this study, since the hypobaric hypoxia-induced decrease in male testosterone was not observed, thereby supporting the hypothesis that the suppression of the gonadal axis in males may occur at very high altitude [8]. Another hypothesis relies on the lower susceptibility of female estrogen axes to hypoxia. The hypoxia-induced decreases in progesterone and estradiol were not statistically significant and were only modest. Our results support the conclusion that dysregulation of the endocrine system at high altitude is only mild-to-moderate [9], by suggesting that up to 5,000 m a.s.l. the changes remain sub-clinical and reversible in both sexes.

Two factors that may have masked the suppression of the male gonadal axis during our expedition may be evoked: (1) the psychophysiological effect linked to the excitement of group and individual success in achieving the objectives of the Himalayan trek, and (2) the stimulating effect of physical activity. Nevertheless, the higher progesterone levels observed in males at peak altitude likely reflect partial inhibition of the steroidogenic pathway that converts progesterone to testosterone, consistent with transient gonadal suppression previously reported in high-altitude expeditions.

To discuss the endocrine system as a whole, the close influence of glucocorticoids and sexual hormones has been widely studied in stress physiology [25, 26], by relying on (1) The pregnenolone steal hypothesis - under conditions of stress, elevated cortisol synthesis reduces the amount of pregnenolone available for the synthesis of downstream hormones other than cortisol - and (2) The HP hypothesis - cortisol suppresses HPG axis diverting resources away from reproduction to prioritise stress responses, by impairing GnRH release which leads to reduced FSH and LH secretion, and consequently reduced hormone production by the gonads. Neither hypothesis seems plausible at high altitudes, given that the cortisol response is typically flat or highly heterogeneous until very high altitudes. Here, the level of the adenohypophysis in the HPG axis was unaffected, implying that hypoxia primarily alters peripheral and regulatory components of the HPG axis rather than directly disrupting pituitary gonadotropin output. Despite the small sample size not permitting the development of a robust model, we state that gonadal axis responses to altitude hypoxia can be studied in both females and males, provided the menstrual cycle is accounted for.

## Limits and perspectives

The main limit of this study is sample size, as the low number of pre-menopausal and non-fertile women inhibits the generalizability of the results. A further limitation of the study design is that it precludes the separation of the effects of exercise from those of altitude hypoxia. The lack of a control group hinders the ability to make inferences regarding the results. Moreover, acetazolamide is known to affect acid-base equilibrium, which may be a confounder in endocrine analysis. Furthermore, it is important to consider the potential effects of jet lag, which can be caused by changes in time zones, dietary habits and sleep patterns. These effects could have affected the impact of altitude hypoxia on endocrine biorhythms.

New studies of females at high altitude could evaluate serial hormone concentrations to assess the real, rather than theoretical, hormonal cycle, thereby creating personalised sex hormone curves through repeated pre-expedition analyses. Moreover, by measuring dopamine and hypoxic ventilatory responses, it will be possible to investigate further the environmental physiology associated with prolactin and progesterone. Possible explanatory insights into the longer ovarian cycle may come from studies of inhibin levels, particularly inhibin B, which is dominant during the follicular phase and may determine the longer duration of this highly variable phase. Complementary analysis of hormones produced by the zona reticularis of the adrenal gland would also provide a more detailed picture of the response of sex hormones. The possible effects of high-altitude trekking on cytochrome P450 17A1 (CYP17A1) enzyme, which has both 17α-hydroxylase and 17,20-lyase activities thus playing a major role in determining steroidogenic pathways, may be studied indirectly with a steroid profiling resulting from product-to-precursor ratios or from a stimulation test.

The implementation of in silico models to estimate short-term fluctuations that govern circadian and infradian hormone rhythms in response to hypoxia could also be a helpful development. Mathematical models have been developed to simulate rhythms of the body’s endocrine network and to incorporate stress parameters [27, 28]. To outline the pulsatility of hormonal axes, specific study designs should be set up to ensure multiple samples within each day of analysis. In a homeodynamic fashion, the spatio-temporal coordination of bodily functions emerges from both top-down and bottom-up mechanisms, and perturbations can alter the output of frequencies and amplitudes across multiple time scales [29]; therefore, hypoxia can be introduced into in-silico models as an environmental perturbation that likely disrupts coherence across time scales.

Finally, analyses of nails and hair may be used to report biomarkers of cumulative hormonal alterations. Both biological matrices can be used to capture long-term hormonal levels by providing a retrospective reflection of endocrine secretion over several weeks or months, thereby overcoming issues associated with single-point measurements, such as limited temporal coverage and intra-individual fluctuations [30, 31].

## Conclusions

Endocrine and metabolic axes and pathways are known to be affected by altitude hypoxia. However, the body’s adaptation to altitude hypoxia is highly heterogeneous across participants and expeditions. Overall, the endocrine system was susceptible to the Himalayas. Within this argument, differential trends by sex emerged for 17β-estradiol, which increased at peak altitude in males; prolactin, whose reduction at peak altitude was clearly observed in females; and progesterone, as males exhibited higher values at high altitude, whereas the average trend was almost flat in females.

The transient perturbations observed in endocrine pathways attest to the organism’s capacity to recalibrate its functional priorities in accordance with principles of efficiency and biological sustainability. When translated into the clinical domain, such a framework offers valuable insights into how the progressive loss of adaptive flexibility—rather than simple quantitative variations in specific hormones—may underlie the pathophysiological core of chronic hypoxia-related disorders.

## Data Availability

The datasets generated during and/or analysed during the current study are available from the corresponding author on reasonable request.

## Declarations

### Generative artificial intelligence (GAI)

No GAI tool was used in the preparation of this manuscript.
